# Correlated biodiversity change between plant and insect assemblages resurveyed after 80 years across a dynamic habitat mosaic

**DOI:** 10.1002/ece3.10168

**Published:** 2023-06-09

**Authors:** Tadhg Carroll, Richard Stafford, Phillipa K. Gillingham, James M. Bullock, David Brown, Michelle Brown, Robin M. Walls, Anita Diaz

**Affiliations:** ^1^ Department of Life and Environmental Sciences, Faculty of Science and Technology Bournemouth University Poole UK; ^2^ Leverhulme Centre for Anthropocene Biodiversity York UK; ^3^ Department of Biology University of York York UK; ^4^ UK Centre for Ecology and Hydrology Wallingford UK; ^5^ National Trust Swanage UK; ^6^ Broadmayne UK

**Keywords:** biodiversity change, community ecology, cross‐taxon correlation, re‐visitation study

## Abstract

Historical data on co‐occurring taxa are extremely rare. As such, the extent to which distinct co‐occurring taxa experience similar long‐term patterns in species richness and compositional change (e.g., when exposed to a changing environment) is not clear. Using data from a diverse ecological community surveyed in the 1930s and resurveyed in the 2010s, we investigated whether local plant and insect assemblages displayed cross‐taxon congruence—that is, spatiotemporal correlation in species richness and compositional change—across six co‐occurring taxa: vascular plants, non‐vascular plants, grasshoppers and crickets (Orthoptera), ants (Hymenoptera: Formicinae), hoverflies (Diptera: Syrphidae), and dragonflies and damselflies (Odonata). All taxa exhibited high levels of turnover across the ca. 80‐year time period. Despite minimal observed changes at the level of the whole study system, species richness displayed widespread cross‐taxon congruence (i.e., correlated temporal change) across local assemblages within the study system. Hierarchical logistic regression models suggest a role for shared responses to environmental change underlying cross‐taxon correlations and highlight stronger correlations between vascular plants and their direct consumers, suggesting a possible role for biotic interactions between these groups. These results provide an illustration of cross‐taxon congruence in biodiversity change using data unique in its combination of temporal and taxonomic scope, and highlight the potential for cascading and comparable effects of environmental change (abiotic and biotic) on co‐occurring plant and insect communities. However, analyses of historical resurveys based on currently available data come with inherent uncertainties. As such, this study highlights a need for well‐designed experiments, and monitoring programs incorporating co‐occurring taxa, to determine the underlying mechanisms and prevalence of congruent biodiversity change as anthropogenic environmental change accelerates apace.

## Graphical abstract

Historical data on biodiversity are rare, particularly for multiple co‐occurring taxa. As such, the extent to which distinct co‐occurring taxa display similar long‐term patterns in species richness and compositional change when exposed to changing environmental conditions is unclear. We present a unique example of correlated biodiversity change in multiple co‐occurring plant and insect taxa resurveyed after 80 years, likely driven by a combination of abiotic and biotic factors.

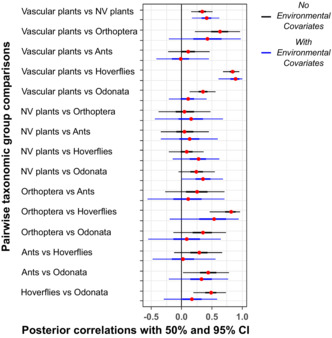

## 1 INTRODUCTION

Biodiversity change, including species losses and gains, is a widespread consequence of changing environments, with poorly understood repercussions for long‐term biodiversity patterns and ecosystem functioning (Harrison et al., 2022; Magurran et al., 2019; Sax & Gaines, 2003; Thomas, 2020; Wardle et al., 2011). In local communities, undisturbed by major human encroachment, losses and gains have led to as much as 10% turnover in species composition per decade (Dornelas et al., 2014, 2019). However, a paucity of historical records has meant the majority of long‐term biodiversity studies have focused on one or very few co‐occurring taxa (but see: Ernest et al., 2008; Ewald et al., 2015; Özkan et al., 2014). As such, it is not clear whether co‐occurring taxa in local communities tend to undergo quantitatively similar biodiversity change when faced with a changing environment. As communities respond to accelerating anthropogenic change, effective conservation planning will require an empirical basis upon which to predict whether disparate co‐occurring taxa will undergo similar changes in species richness and composition.

Ecological communities commonly display cross‐taxon congruence—correlated patterns in species richness or composition—over spatial extents (Westgate et al., 2014), though the strength of congruencies is variable, depending on the taxa involved and spatial scale of analysis (Pearson & Carroll, 1999). Cross‐taxon congruence in *temporal* richness and compositional change may occur also, due to a range of abiotic and/or biotic drivers. Abiotic drivers play a dominant role underlying temporal biodiversity change across a range of taxa and systems (Avolio et al., 2021; Feeley et al., 2020; Mutshinda et al., 2009). Environmental change should therefore underlie congruent biodiversity trends in co‐occurring taxa with similar abiotic requirements. Interactions between taxa—particularly those with strong trophic or mutualistic dependencies—may also play a key role, with plant richness and composition in particular often associated with biodiversity in higher taxa (e.g., Scherber et al., 2010). Thus, biodiversity change in interacting taxa may induce positive or negative change in interaction partners depending on the nature of interaction (Kiers et al., 2010; Pace et al., 1999). Gaining a better understanding of how such processes play out across trophic levels should be an important next step in research on biodiversity change.

**FIGURE 1:**
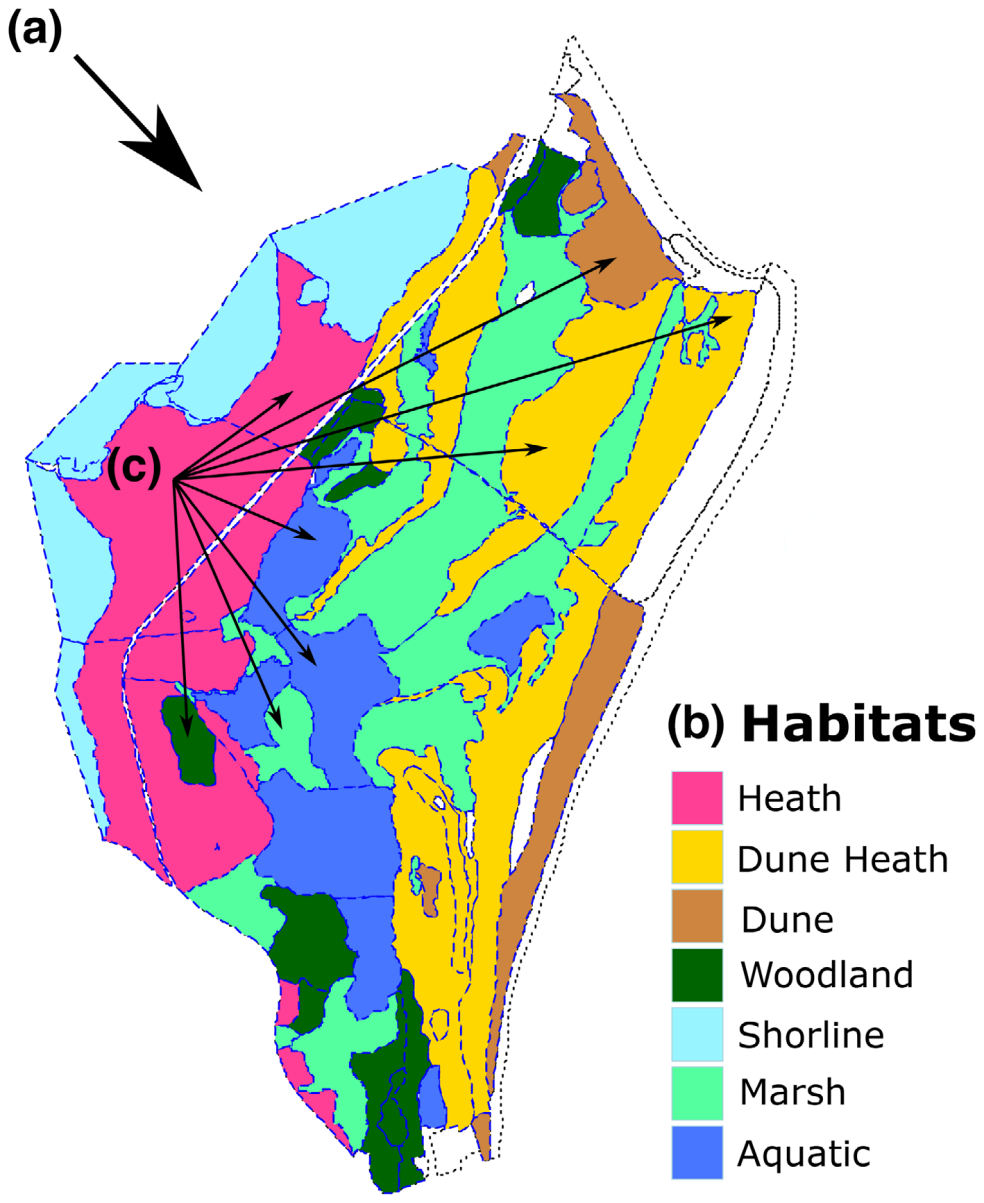
Studland peninsula in Dorset consists of a mosaic of ecological habitats surveyed in the 1930s and 2010s. Dashed blue lines separate the 74 “sampling compartments” of historical and contemporary surveys, and colors denote habitat types. Even within this small peninsula (~3 km^2^), temporal biodiversity change in plant and insect communities can unfold in differing ways at distinct spatial scales: (a) changes at the level of the whole study system; (b) changes within distinct ecological habitat types; (c) changes within and among sampling compartments. Species richness and compositional changes may correlate across taxa (cross‐taxon congruence) at any or all scales due to shared responses to a changing environment and/or biotic interactions.

Here, using a dataset unique in its combination of temporal and taxonomic scope, we investigated whether local plant and insect communities—surveyed in the 1930s and resurveyed in the 2010s—displayed cross‐taxon congruence in species richness and compositional change between time periods. We assessed temporal congruence at three nested local scales (Figure 1), across six taxa representing distinct evolutionary histories, ecological requirements, and trophic characteristics (Table 1). To quantify temporal cross‐taxon congruence and its probable drivers, we asked: (i) To what extent have species richness and composition in each taxonomic group changed between sampling periods, and how do changes compare across taxa?; (ii) What environmental factors best predict species composition within each taxon during each sampling‐period, and have composition/environment relationships changed between sampling periods?; (iii) Do plant and insect taxa display temporal congruence in local species richness change among assemblages?; (iv) To what extent is congruence between taxa in species richness change associated with shared responses to environmental change?

**TABLE 1:** Focal taxa and their trophic status.

Taxon	Trophic status
Vascular plants	Primary producers
Non‐vascular plants	Primary producers
Orthoptera (Grasshoppers/Crickets)	Herbivores
Hymenoptera Formicidae (Ants)	Omnivores (herbivorous/carnivorous, diet varies by species)
Diptera Syrphidae (Hoverflies)	Larva: Herbivores/Carnivores (diet varies by species)
	Adult: Herbivores (strong dependency on nectar/pollen)
Odonata (Dragonflies/Damselflies)	Carnivores

We hypothesized that: (i) changes in composition of herbivore communities would correlate more closely with change in plant community composition than would changes in omnivores and carnivores, and (ii) congruencies observed in pairwise comparisons among the four non‐plant taxa would be driven largely by shared responses to abiotic change, as no strong trophic or structural dependencies exist between these groups.

## 2 METHODS

### 2.1 Study site and data collection

The study was conducted on the Studland peninsula at the south‐eastern edge of Poole Harbor in Dorset, England (Lat: 50.66 N, Lon: 1.9 W). The peninsula consists of a diverse mosaic of ecological habitats (Lowland Heath (Wet and Dry), Dune heath, Dune, Woodland, Marsh, Shoreline, and Aquatic (edge)), and covers approximately 3 km^2^ (Figure 1). The broad change in the habitats of the peninsula over the 80 years between sampling periods has been succession from unvegetated blown sand to heathland and scrub on the Studland Bay side of Ferry Road. The older heathland found mostly on the harbor side of the road has seemingly changed little, though a large increase in public pressure has likely had its effects. The younger dunes and slacks of the 1930s are now mature and a completely new dune has become established since Diver's 1930s survey (below). The succession is clear from the change in EIV scores for light (L) and pH (R) (Carroll et al., 2018; R. M. Walls, pers.comm.). The inference is that the open vegetation typical of sand dunes has closed and nutrients are accumulating in soil as it develops from loose sand.

Between 1933 and 1938, the naturalist Cyril Diver led extensive surveys of Studland in his spare time, documenting occurrences of a wide range of plant and insect species (Diver, 1938; Diver & Diver, 1933; Good, 1935). Diver and his group meticulously recorded species' occurrences onto specially designed ordinance survey maps (Figure S1; Diver & Good, 1934), broken down into “Locus‐Habitats,” which were designated as being relatively homogenous based on the topographical properties and local ecological characteristics of the peninsula (Carroll et al., 2018; Diver, 1938). The National Trust, in collaboration with volunteer naturalists, later delineated Diver's Locus‐Habitats into 74 “sampling compartments” for targeted searches, based on Diver's map and notes, and on physical features of the peninsula (Figure 1). Compartments varied in shape, size, and habitat designation (min = 900 m^2^, max = 200,764 m^2^, mean = 44,453 m^2^; Data S1). Diver's species lists and maps were used to compile historical species lists for each compartment, for each taxonomic group.

The area was resurveyed by a combination of experienced amateur and professional naturalists in an initiative led by the National Trust between 2013 and 2015 (“The Cyril Diver Project”). The resurvey effort recorded species occurrences at the level of sampling compartments. Surveys targeting each taxonomic group were led by experts with respect to that group in each time period, and sampling methods differed depending on which group was being targeted. Visual searches and the compilation of incidental records were common across all taxa in both time periods. Quadrats placed randomly within compartments were also employed in surveys for vascular and non‐vascular plants. Sweep netting and pitfall traps were used in insect surveys for both time periods, and occasional malaise traps and vacuum samples were also used in the 2010s.

While it was not possible to replicate sampling methods to exactly match the 1930s surveys (in terms of sampling effort and precise methods deployed), both historical and contemporary surveys went to great effort to record the full set of species in each taxonomic group across each sampling compartment for which that taxon was surveyed. The 2010s survey had the advantage of being conducted throughout much of the year whereas 1930s surveys were concentrated in the summer holidays. Moreover, during the 2010s, resurveyors had compartment‐level species lists from the 1930s, which allowed targeted searches for species known to have occurred in a particular area in the 1930s that had not yet been found. As such, it is possible that inferred species losses may be more reliable than species gains in some instances, as Diver did not have the luxury of the contemporary species lists with which to target searches (but see Sections 2.4 and 3.5 below).

It is very difficult to gauge the difference in level of identification skills between groups. The current wealth of identification books and the internet were not available in the 1930s. A case in point is the publication of the Vegetative Flora (Poland & Clement, 2009) which enables reliable identification when flowers are not present on the specimen because of the season or loss through grazing. Another difference, that may have a minor impact on the conclusions in this paper, is the advance in taxonomic treatment of the group. Nomenclature changes are easily resolved, but where a species has been split the resurvey may appear to be more species‐rich. Examples, again in the area of vascular plants, is the circumscription of the yellow sedges (*Carex flava* aggregate), and birch, which Diver recorded as *Betula alba*, is now recognized as two species. Fortunately, only one of the species (*Betula pendula*) is common on most of the peninsula. Straightforward misidentification is possible at any time and its impact is essentially imponderable.

Analyses of biodiversity change for the six focal taxa (Table 1) were based on differing subsets of sampling compartments for each taxon. The baseline set of sampling compartments used for analyses of biodiversity change included the 74 compartments for which reliable vascular plant species lists were available for both sampling periods (depicted in Figure 1). Species lists for each of the other 5 taxa were available for differing subsets of the baseline sampling compartments (vascular plants (*n* = 74), non‐vascular plants (*n* = 45), grasshoppers and crickets (*n* = 39), ants (*n* = 48), hoverflies (*n* = 34), and dragonflies and damselflies (*n* = 53)), as reliable lists were not available for all taxa in all compartments (Data S1). In some instances, species lists were unavailable because the focal taxon was unlikely to inhabit the compartment, particularly for compartments in the aquatic habitat. In other cases, historical lists were missing, or the compartment was not heavily targeted for sampling under the more recent survey, and thus meaningful biodiversity comparisons could not be made.

### 2.2 Environmental data

Between sampling periods in the 1930s and 2010s the peninsula underwent considerable and heterogeneous change, likely driven by hydrological differences, vegetative succession, and nitrogen deposition (Carroll et al., 2018). Environmental data used in statistical analyses consisted of categorical variables describing the ecological habitat type (Figure 1), and compartment mean Ellenberg Indicator Values (EIVs) for both time periods for moisture (F), Light (L), Soil Nutrients (N), pH (R), and Salinity (S) derived from hierarchical regression models (Carroll et al., 2018). EIVs provide a species‐specific score which grades plant species according to observed environmental associations (Ellenberg, 1988; Hill et al., 2004). Site mean EIVs are widely used in re‐visitation studies as proxies for alternative environmental drivers in the absence of directly measured environmental data (e.g., Diaz et al., 2013; Newton et al., 2012).

### 2.3 Statistical analyses

#### 2.3.1 Compositional change between time periods (species losses and gains)

**FIGURE 2:**
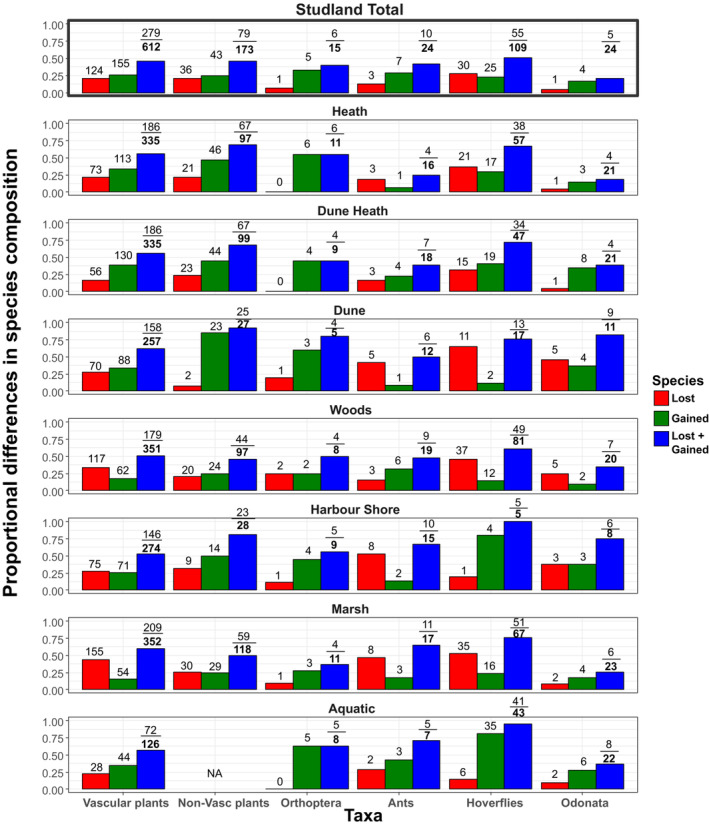
Between time period compositional change for six plant and insect taxa within ecological habitat types and for the whole Studland peninsula for sampling periods in the 1930s vs. 2010s. Proportional differences (*y*‐axes) in species composition (blue) are computed as the number of species losses (red) + gains (green) as a proportion of the total number of species present in either or both time periods combined, and are identical to the Jaccard dissimilarity index computed between two time periods. A value of 1 on the *y*‐axis for blue bars indicates that the identity of every species recorded in the assemblage has changed between sampling periods, while a value of 0 indicates that exactly the same species were recorded in each time period. Numbers labelling the bars are the raw species counts for each sampling unit from which the proportions were derived. Change in species richness can be calculated as gains minus losses in each instance.

To quantify changes in species richness and composition between sampling periods at the levels of the whole study system and ecological habitat types, we used the Temporal β‐diversity Indices (TBI) of Legendre and Salvat (2015). TBI are a simple but intuitive and information rich method based on breaking down components of the classical dissimilarity indices used in community ecology—in this case the Jaccard similarity index. For presence/absence data this amounts to simply counting the number of species losses and gains between two sampling periods in a sampling unit (whole peninsula, habitat), and calculating the sum of species gains plus losses as a proportion of the total number of species present in either or both time periods (losses + gains + species found in both time periods) to find the proportional change in species composition between sampling periods. We calculated TBI for each taxonomic group within each ecological habitat type that had compartment‐level species lists for that group, and for the peninsula as a whole (for a total of 47 calculated metrics). We used these to compare proportional changes in community composition between sampling periods across taxa and how these proportions were broken down by species losses and gains in the raw data (Figure 2). We also calculated observed species richness differences for each sampling unit.

#### 2.3.2 Species composition in relation to the environment

To identify environmental factors underlying compositional differences between the 1930s and 2010s, we assessed relationships between species composition and environmental conditions for each taxon within each sampling period and how these relationships changed between sampling periods. To quantify composition/environment relationships, we performed redundancy analyses (RDA) and variation partitioning on multivariate Hellinger transformed species‐by‐compartment occurrence matrices for each taxon within sampling periods 1 and 2 (Legendre & Gallagher, 2001; Legendre & Legendre, 2012). RDA is a form of constrained ordination analogous to multivariate regression, which allows the user to quantify proportions of explained variance in species composition (adjusted *R* ^2^) attributable to continuous or categorical predictor variables across a set of sampling units (Legendre & Legendre, 2012).

We fitted RDAs for each of the six focal taxa using two sets of environmental explanatory variables across the sampling compartments: (1) broad ecological habitat types as categorical predictors, and (2) mean within sampling‐period EIVs for moisture (F), Light (L), Soil Nutrients (N), pH (R), and Salinity (S) (Carroll et al., 2018). EIVs for nutrients (N) and pH (R) were highly collinear across sampling compartments, so final models only included EIV R (not EIV N) as EIV R explained more variance (by adjusted *R* ^2^ criteria) in almost all cases. We selected statistically significant EIV predictors at the alpha = .05 level for each taxon using the forwardsel function from package VEGAN in R (Oksanen et al., 2013). Finally, we used multivariate variation partitioning with VEGAN's varpart function for each set of models to determine the shared and unique contributions to the explanation of variation in species composition attributable to habitat type versus EIV explanatory variables.

#### 2.3.3 Cross‐taxon congruence in pairwise comparisons of species richness change

To quantify temporal cross‐taxon congruence in species richness change between sampling compartments—that is, the magnitude of correlations in richness differences between pairs of taxa—we used a hierarchical logistic regression (HLR) based approach. We first extracted just the species lost and gained between sampling periods for each compartment and coded them as 0 and 1 respectively. We fitted a HLR with intercepts varying by compartment for the vascular plant taxonomic group, and five separate sets of slope parameters, also varying by compartment, for each of the other five focal taxa (coded as dummy variables) (Model M1, Data S2). We included a random effect for species ID to ensure we estimated the average change in species within taxa for comparison. We reconstructed intercepts from the fitted model for each taxon on the inverse logit scale, corresponding to the proportion of species gained versus lost in each sampling compartment averaged over species. This proportion provided us with a proxy for species richness change that we could compare across taxa: a proportion greater than 0.5 represents an increase in richness, while a proportion less than 0.5 represents a decrease. We then computed Pearson's correlations of the intercepts between each pair of taxa over posterior distributions of estimates from the models across the subset of compartments with records for both taxa within a pair. This provided a measure of cross‐taxon congruence for average richness change between taxa.

#### 2.3.4 Congruence due to shared responses to environmental change

To assess whether correlations in species richness change between taxa were likely driven by shared responses to environmental change, we fit a second hierarchical logistic regression model (Model M2, Data S2), extending the variable intercept and slope parameters of M1 to include predictors for habitat type and for the change in mean EIVs between sampling periods. The inclusion of habitat type to represent environmental change entails the assumption that environmental conditions in distinct habitat types changed in different ways from one another between sampling periods, and this is indeed the case (Carroll et al., 2018).

**FIGURE 3:**
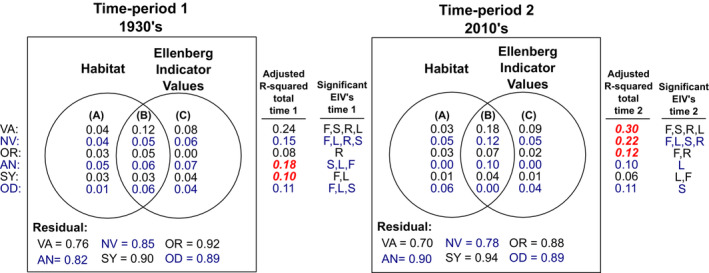
Relationships between species composition and the environment across the Studland peninsula derived from Canonical Redundancy Analyses (RDA) and multivariate variation partitioning performed separately on vascular plant (VA), non‐vascular plant (NV), grasshopper and cricket (OR), ant (AN), hoverfly (SY), and dragonfly and damselfly (OD) assemblages for sampling periods in the 1930s and 2010s. Venn diagrams display proportions of variation explained (adjusted *R* ^2^) by: (a) ecological habitat type alone, (b) jointly by habitat type and site mean Ellenberg indicator values (EIVs), and (c) EIVs alone. EIVs F, L, R, and S represent soil moisture, light availability, pH, and salinity, respectively. Only significant EIVs included in the final models were used for calculations of adjusted *R* ^2^. Red numbers indicate that more variation was explained by environmental predictors in that time period for a taxonomic group.

The additional predictor terms were included as varying offsets for habitat type with varying slopes for change in EIV scores and were fitted using a multivariate normal distribution with an associated variance/covariance matrix and with a non‐centered parameterization to aid model fit (McElreath, 2016a; Stan Development Team, 2018a). EIV predictors included in this model were those found to be significant predictors of community composition from RDA analyses (above) for each taxon (see Figure 3), so that we were only using environmental covariates found to have an association with assemblage composition for a specific taxon, and to help avoid overfitting. Pairwise correlations were then recomputed over posterior distributions having adjusted for these components of environmental change. Hierarchical models were fitted with MCMC in Stan using the rethinking package in R version 3.5.0 with four parallel chains of 4000 iterations each (McElreath, 2016b; R Core Team, 2018; Stan Development Team, 2018b). Details of fitted models including prior distributions can be found in Data S2.

### 2.4 Data and model validation

We deployed a suite of data and model validation techniques to assess the risk of biases arising due to comparisons between historical and contemporary datasets. To assess sample “completeness” during the contemporary (2010s) survey period, we used the “specaccum” function from the R package “vegan” (Oksanen et al., 2013), fitting species accumulation curves to raw data for each taxonomic group at the levels of ecological habitat type and the whole peninsula (Data S3). Unfortunately, this was not possible for the 1930s survey period due to a lack of temporal resolution within historical data. However, we also used a Raup‐Crick null model‐based approach to assess whether changes in the composition of compartment‐level assemblages across time periods were different from changes that would be expected by chance (Chase et al., 2011), in particular, contrasting within‐ versus between‐compartment compositional differences (Data S4). Findings of more similar species composition within compartments (between time‐periods) than across compartments would provide reassurance that historical and contemporary data could provide meaningful comparisons, though major compositional change between time periods could introduce some uncertainty under this method. We also use the Raup‐Crick null models to test whether size differences between sampling compartment biased estimates of compositional change.

To further assess the possibility that sampling bias or bias introduced due to effects of rare, cryptic, or transient species adversely affected our findings, we refitted hierarchical models M1 and M2 including only species loss/gains for species observed in 20% or more sampling compartments during the time period in which a species was more widespread (taxon‐specific). We then re‐computed the cross‐taxon correlations (described in Sections 2.3.3 and 2.3.4) to check if they differed substantially from correlations computed under the full dataset. This sensitivity analysis should serve to minimize effects of rare, cryptic or “vagrant” species on cross‐taxon correlations, and assess whether only species recorded as lost or gained that were legitimately present in one time period and not the other influenced inference under this analysis. We also tested for spatial autocorrelation in R by computing “Moran's I” values on an adjacency matrix of sampling compartments (following Brunsdon & Comber, 2018) for compartment‐level proportional gains versus losses of species for each taxonomic group, as well as for hierarchical model estimates of the same proportions and compartment‐level binned model residuals (taxon‐specific; Data S5). Relatively small sample sizes precluded the addition of spatial covariates to RDA models. Posterior predictive checks were also performed for hierarchical logistic regression models (Data S6).

## 3 RESULTS

### 3.1 Compositional change between time periods (species losses and gains)

All six taxa displayed large proportional differences in species composition between sampling periods, both across the peninsula as a whole, and within ecological habitat types (Figure 2). Peninsula‐wide differences in the composition of recorded species were of a similar order of magnitude across taxa (proportional difference in species composition ≈ 0.5, with the exception of Odonata (proportional difference = 0.24)), but proportions of species losses versus gains which contributed to differences were less uniform across the groups. Orthoptera, ants, and odonates, in particular, showed larger gains than losses (Figure 2). These groups have much smaller total species numbers than the other three groups, and as such, their full cohorts of species were likely more completely sampled (Data S3). At the level of ecological habitat types, compositional differences were variable both within and between taxa, but the total differences tended to be larger than at the whole peninsula level.

The number of species recorded increased marginally at the study system level for all taxa apart from hoverflies (net loss of 5 species). Total net species gains or losses were composed of gains in some ecological habitat types and losses in others for five of the six taxa (excluding Orthoptera which exhibited no net losses; Figure 2). In general, recorded gains dominated in the heath, dune heath, harbor shore, and aquatic habitats, while losses tended to be more prominent in the wood and marsh habitats.

### 3.2 Species composition in relation to the environment

Composition/environment relationships displayed widespread changes for all six taxa between sampling periods (Figure 3). The most influential factors in predicting species composition across taxa at compartment level within each sampling period were wetness and light availability (EIVs F and L), along with ecological habitat type. Variation partitioning revealed that habitat type and EIVs shared a large amount of explained variation for models of all taxa in time period 1 and for five of the six taxa in time period 2 (excluding Odonata). Explained variance (adjusted *R* ^2^) increased in the 2010s versus the 1930s for vascular plants, non‐vascular plants and Orthoptera (by 0.07, 0.07, and 0.04, respectively)—mostly explained jointly by habitat type and EIVs, rather than being exclusively explained by either set of predictors. This indicates that the composition of recorded species differed more among habitats in the 2010s for these groups, possibly due to a greater contrast in environmental conditions across habitats compared with the 1930s. Adjusted *R* ^2^ for ant and hoverfly groups showed decreases between sampling periods in the 2010s versus the 1930s (by 0.08 and 0.04, respectively). Both groups exhibited a shift toward variance explained jointly by habitat and EIVs in the latter time period.

### 3.3 Cross‐taxon congruence in pairwise comparisons of species richness change

**FIGURE 4:**
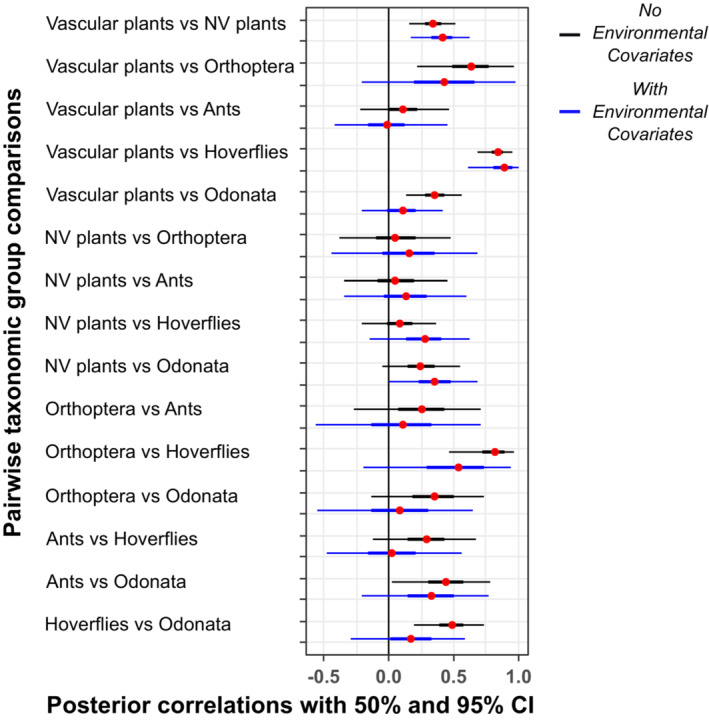
Pearson correlations between pairs of taxa in average proportions of species gained versus lost across the sampling compartments of the Studland peninsula between the 1930s and 2010s. Black credible intervals are pairwise correlations derived from a hierarchical logistic regression model including a varying compartment level intercept for vascular plants, varying slopes for the other five taxa, and a random offset for species ID. Blue credible intervals are pairwise correlations from the same model extended to include habitat and environmental change covariates, aiming to give residual correlations after adjusting for shared responses to environmental changes between time periods. Correlations were computed over the full posterior distribution of Bayesian models with plot showing medians (red dots), 50% and 95% credible intervals (thick and thin lines, respectively).

Hierarchical logistic regression models revealed strong evidence for cross‐taxon congruence in temporal species richness change across the study area, illustrated by pairwise correlations between taxa in Figure 4. Median pairwise correlations between taxa in species richness change were non‐negative in all cases before accounting for environmental change, and the 95% credible intervals were fully positive (did not cross zero) in 7 of the 15 comparisons (analogous to frequentist statistically significant congruencies at the α = .05 level (Chen et al., 2013), black intervals Figure 4). Correlations between vascular plants and other taxa (excluding ants) were strong and positive, and correlations between vascular plants and the two herbivorous groups (hoverflies and Orthoptera) were stronger than those with non‐herbivores. Non‐vascular plants displayed the weakest correlations with other taxa, with the only notable correlations between them and vascular plants, and a weaker (median ≈ 0.25) correlation with Odonata. Pairwise correlations between insect groups were broadly positive, with three out of six of the distributions “statistically significant” before accounting for environmental change.

### 3.4 Congruence due to shared responses to environmental change

When environmental covariates were adjusted for, 8 of the 15 pairwise correlation distributions shifted in a negative direction, including all six insect–insect group comparisons (Figure 4, blue intervals). Correlations between vascular plants and herbivores stayed strong and positive, but the correlation between vascular plants and Odonata shifted to center at approximately zero. Correlations between non‐vascular plants and all other groups shifted in a positive direction but remained weak in all comparisons apart from correlations with vascular plants and Odonata.

### 3.5 Data and model validation

Results of species accumulation curves and Raup‐Crick null model analyses suggest that data from contemporary and historical survey periods were reasonably complete and comparable (Data S3 and S4, respectively). Species accumulation curves at the whole peninsula and habitat levels for contemporary (2010s) sampling suggest that species richness and composition were suitably well estimated for this sampling‐period (Data S3). As such, we can have good confidence that inferred species losses were reliable at these hierarchical levels. Since we could not compile similar accumulation curves for historical data, we cannot rule out the possibility that species gains may have been overestimated. However, results from Raup‐Crick null models show that within‐compartment differences in community composition between time periods skewed toward being markedly more similar than would be expected by chance (and more similar than comparisons across different compartments) for four of the six taxa, providing evidence that between time period sample comparisons are justified (Data S4). Cross time period comparisons were more different than expected by chance for hoverflies and were approximately equally likely to be more similar or more different for ants (possible reasons are discussed in Section 4). No taxa showed any association between compartment size and cross time period differences (Figure S11).

Both of the hierarchical regression models fitted to the subsampled dataset retaining only species present in 20% or more compartments produced cross‐taxon correlations that were strikingly similar to models fitted using the full dataset. This is likely due to the species‐level random effect, which means that correlations were computed for an average species within a taxonomic group, and therefore rare and transient species will not have made large contributions to estimated correlations. For this reason, correlations should be interpreted as being more strongly influenced by commonly observed species across taxa. Despite some spatial autocorrelation in patterns of species losses and gains across the study area, we found minimal evidence of spatial autocorrelation in compartment‐level binned model residuals (Data S5), providing further evidence that hierarchical model estimates of cross‐taxon congruence are sound (Kühn & Dormann, 2012).

## 4 DISCUSSION

The plant and insect assemblages of Studland displayed strong evidence of cross‐taxon congruence in species richness and compositional change across the approximately 80‐year period. All taxa exhibited high levels of turnover within habitat types and across the peninsula as a whole. Observed species richness did not change substantially for any group at the study system level. However, hierarchical model estimates suggest that shared responses to environmental change may have contributed to widespread congruencies observed in richness differences across local assemblages within the study system. Biotic interactions between plants and herbivores were also identified as plausible drivers of correlated species richness change, as correlations were strongest between vascular plants and their consumer groups and were largely unaffected by adjusting for environmental change.

High levels of compositional change observed at Studland are in line with findings from synthesis studies of local biodiversity trends over this period across a wide range of taxa and systems (Dornelas et al., 2014; McGill et al., 2015). Proportional turnover was remarkably similar for five of the six taxa at the study system level, reflecting findings of cross‐taxon congruence in species composition across spatial extents in contemporary communities (Westgate et al., 2014). Compositional differences were much more variable within ecological habitat types than at the study system level, highlighting the importance of scale and habitat classification in studies of cross‐taxon congruence (Schuldt et al., 2015; Westgate et al., 2014).

Despite widespread turnover, observed species richness differences were minimal for all taxa at the study system level. This finding is typical of local biodiversity trends in undisturbed systems (Dornelas et al., 2014, 2019; Vellend et al., 2013), where long‐term regulation of species richness may be a widespread phenomenon (Brown et al., 2001; Gotelli et al., 2017). However, species richness change computed from the raw data at this scale may be subject to biases (see Section 2 and further discussion below) and should therefore be interpreted with caution. However, congruent species richness differences were also widely prevalent in pairwise comparisons between taxa across sampling compartments, as estimated from hierarchical models.

Few other studies have investigated temporal cross‐taxon congruence in biodiversity change, though it has been found when looked for. Ewald et al. (2015) found that extreme weather events had comparable inter‐annual effects on abundances in 11 out of 26 insect taxa in a 42‐year time series recorded at genus, family, and class level in cereal fields. They also found correlations in temporal abundance trends with temperature and precipitation, and detrimental effects of pesticide use across taxa. Özkan et al. (2014) reported temporal congruence between phytoplankton and zooplankton, apparently driven by environmental factors and trophic interactions. It should also be noted that biodiversity change can occur in a time‐delayed manner, with some taxa responding faster than others to environmental drivers (Daskalova et al., 2020), which may have important knock‐on effects via biotic interactions (Valiente‐Banuet et al., 2015).

As in other documented cases of congruent temporal changes across taxa (Ewald et al., 2015; Özkan et al., 2014), changes in the abiotic environment likely played a role underlying observed compositional changes at Studland. Soil moisture (EIV F) and light availability (EIV L) were important predictors of species composition across taxa within sampling periods, and the study system has undergone widespread hydrological change and varying degrees of vegetative succession between the sampling periods (Carroll et al., 2018). Abiotic factors are important drivers of biodiversity change in general (Mutshinda et al., 2009; Vellend, 2016), as species adapted to particular environmental conditions respond to environmental change (Carroll et al., 2023), and hydrology and vegetation structure in particular are both important determinants of plant and invertebrate community composition (De Szalay & Resh, 2000; Silvertown et al., 2015). Effects of environmentally induced selection were further supported by congruent species richness differences at the sampling compartment level, where adjusting for environmental factors broadly reduced model estimates of temporal congruence in species richness differences.

RDA models explained only small proportions of variation in species composition across most insect groups in each time period. Any number of unmeasured environmental factors could be responsible for the remaining unexplained variation, including effects of microclimate (Gillingham et al., 2012), grazing patterns of deer and cattle (Diaz et al., 2005; Newton et al., 2009) and variable human use intensity across sampling compartments. Proportions of variation explained by the categorical habitat type variable also represent a broad suite of abiotic and biotic aspects of the underlying environment, which will not have been captured in full by the RDA models. Given the hydrological, topographical, and geological characteristics of Studland (Diver, 1933), as well as the nature of the sampling regime, it is also possible that spatial autocorrelation adversely affected RDA estimates (e.g., with respect to *p*‐values or effect sizes), as relatively small sample sizes precluded the addition of spatial covariates. However, spatial autocorrelation did not affect our primary finding of cross‐taxon congruence in local species richness change. This can be seen from residuals of the hierarchical logistic regression models, which display minimal evidence of spatial autocorrelation despite autocorrelated patterns in raw proportions of species gains versus losses (Kühn & Dormann, 2012; Data S5).

Vascular plants displayed strong congruencies in species richness differences at sampling compartment level with all taxa except ants. Moreover, pairwise congruencies were stronger between vascular plants and herbivores (hoverflies and Orthoptera) than with non‐herbivore insect groups, as hypothesized. This finding suggests a role for bottom‐up effects of the plant community on composition of at least these herbivore groups—a finding in‐line with previous experimental and observational results (Rzanny et al., 2013; Schaffers et al., 2008; Scherber et al., 2010). However, the possibility of top‐down causation, or a combination of top‐down and bottom‐up cannot be ruled out.

The relationship between vascular plants and hoverfly richness differences was particularly strong and echoes the national trend over this time period (Biesmeijer et al., 2006). In addition, null model results showed that within‐compartment hoverfly assemblages were, in general, more likely to differ across time periods than would be expected by chance (and more likely to differ than other taxa were; Data S4), possibly suggesting strong drivers of change for this group. Adult hoverflies feed exclusively on nectar and pollen from flowering plants and some species have associations with specific vegetation types while attempting to attract mates (Gilbert & Rotheray, 2011). Larva of many species also have trophic associations with particular plant species, either feeding on them directly or searching for invertebrate prey which feed upon them (Almohamad et al., 2009). Some plant species may also rely on hoverflies as pollinators (Rader et al., 2016). Due to the number of potential pathways underlying this correlation, more work is needed to determine the nature of underlying mechanisms.

Non‐vascular plants and ants were outliers to some extent in analyses of biodiversity change. Accounting for environmental factors increased congruence estimates between non‐vascular plants and all other groups. Many non‐vascular plant species thrive in wetter conditions (Silvertown et al., 2015) and increases in soil moisture across large swathes of the peninsula (Carroll et al., 2018) may have increased species richness in this group while largely excluding species across other taxa. For ant assemblages, RDA models described far more variance in composition in the 1930s than in the 2010s (0.18 and 0.1 respectively), and they were also the only taxonomic group not to display any congruence with vascular plant changes. Widespread gains across the study area made by the now dominant ant species *Formica rufa* may go some way to explain the idiosyncratic trend in this group, as competitive and predatory effects of this species may have had overwhelming effects on ant community structure through dominance hierarchy effects (Halaj & Wise, 2001). However, null model dissimilarity estimates across time periods for Ants (within sampling compartments) were equally likely to be more or less different than would be expected by chance, further highlighting differences with other taxa (Data S4). This could be due to the hypothesized effect of *Formica rufa* but could also hint at possible sampling issues in this group. As such, conclusions should perhaps be accepted with a greater degree of skepticism for ants than for other taxa.

Biodiversity change is unusually well documented for the plant communities of Dorset over the time period of this study (Diaz et al., 2013; Hooftman & Bullock, 2012; Jiang et al., 2013; Keith et al., 2009, 2011; Newton et al., 2012), and Studland is situated in a rare remaining biodiversity “hotspot” (Jiang et al., 2013). Dorset has seen a significant decline in α‐diversity (local richness) of plants on average due to widespread habitat loss and agricultural intensification (Jiang et al., 2013). However, within remaining local patches, woodland, lowland heath, and calcareous grasslands have seen variable patterns of richness and compositional change, with each habitat experiencing idiosyncratic drivers of change between sampling periods (Diaz et al., 2013; Keith et al., 2009, 2011; Newton et al., 2012). Similar data are not available for the insect taxa of Dorset over this period. However, our results suggest that the patterns of richness and compositional change documented in Dorset's plants are likely to have coincided with congruent changes in co‐occurring insect assemblages.

When comparing data collected under non‐identical sampling regimes, biased estimates of ecological phenomena are an inherent danger (e.g., because of differences in sampling effort causing rare or cryptic species to be differentially represented (Chen et al., 2013), or spatiotemporal variance in sampling effort (Fithian et al., 2015)). Due to unavoidable differences between survey methods in each time period (see Section 2.1 above), and because we could not compile species accumulation curves for historical data, we cannot rule out for certain the possibility that species gains were overestimated. However, by refitting hierarchical models for species present in 20% or more sampling compartments we have at least partially mitigated similar concerns with respect to bias arising from sampling issues at this scale, as cross‐taxon correlations computed from these models were very similar to models under the full dataset, due to species‐level random effects giving less weight to rare and transient species and estimating correlations between the “average” species within taxa (Gelman & Hill, 2006).

Assessing the relative contributions of different drivers of biodiversity changes can be difficult, even with good estimates of environmental change at hand, as positively or negatively correlated responses can mask the true nature of relationships (Ranta et al., 2008). The hierarchical modeling framework used here also helps circumvent this issue by modeling separate intercept and slope parameters for each taxon with associated predictors of taxon‐specific effects of environmental change. Null model analyses further suggested that comparisons of compartment‐level assemblages across time periods were meaningful, as within compartment assemblages tended to be more similar than would be expected by chance, and more similar than comparisons across differing compartments (Data S4; but see discussion of ants and hoverflies above). Nonetheless, if between time period sampling effort differed among sampling compartments in a consistent manner across taxa, spurious cross‐taxon correlations in species loss/gain patterns remain a possibility we cannot rule out.

We have shown that congruent biodiversity change occurs across diverse plant and insect taxa and is likely driven by changes in both abiotic and biotic environmental conditions. These drivers do not act independently from one another, as demonstrated by likely effects of biotic interactions between the plant community and herbivores, while both taxa appeared also to respond to abiotic change. These results suggest that the wide ranging anthropogenic environmental changes projected to continue throughout the 21st century may have comparable effects on species richness and composition across plant and insect taxa co‐occurring in local communities. However, comparisons involving historical data of this nature come with inherent uncertainties, even more so than for ordinary observational comparisons. As such, a strong implication of our findings is the need for carefully designed experiments, and monitoring programs encompassing multiple co‐occurring taxa, to uncover mechanisms underlying temporal congruence in biodiversity change and to estimate the prevalence with which such trends occur in nature.

## AUTHOR CONTRIBUTIONS

**Tadhg Carroll:** Conceptualization (lead); data curation (lead); formal analysis (lead); investigation (lead); methodology (lead); project administration (lead); validation (lead); writing – original draft (lead); writing – review and editing (lead). **Richard Stafford:** Conceptualization (supporting); investigation (supporting); methodology (supporting); supervision (equal); writing – review and editing (supporting). **Phillipa K. Gillingham:** Conceptualization (supporting); investigation (supporting); methodology (supporting); supervision (equal); writing – review and editing (supporting). **James M. Bullock:** Supervision (supporting); writing – review and editing (supporting). **David Brown:** Conceptualization (supporting); data curation (lead); funding acquisition (lead); investigation (supporting); supervision (supporting). **Michelle Brown:** Conceptualization (supporting); data curation (lead); investigation (supporting). **Robin M. Walls:** Conceptualization (supporting); data curation (supporting); investigation (supporting); writing – review and editing (supporting). **Anita Diaz:** Conceptualization (supporting); funding acquisition (lead); investigation (supporting); supervision (lead); writing – review and editing (supporting).

## CONFLICT OF INTEREST STATEMENT

The authors declare no conflict of interest.

## Supporting information


Data S1:

## Data Availability

Data can be found in Dryad repository (https://doi.org/10.5061/dryad.547d7wmdn).
