# Supplementary material for: Correlated biodiversity change between plant and insect assemblages resurveyed after 80 years across a dynamic habitat mosaic
Source: Ecol Evol. 2023 Jun 9;13(6):e10168. doi: 10.1002/ece3.10168 (PMC10251423; doi:10.1002/ece3.10168)
Supplement: Supplementary file 1 — Data S1: [file ECE3-13-e10168-s001.docx]

# Supplementary materials for Congruent change in plant and insect assemblages over 80 years across a dynamic habitat mosaic.

Figure S1 Diver’s original “locus habitats” (left) and the latterly compiled “sampling compartments” (right) of Studland.

## S1 | Taxon-specific compartment lists and compartment characteristics

Table S1 Table of the “sampling compartments” of the Studland peninsula. “c.n” are numerical identifiers for sampling compartments, for which there are separate columns to specify whether or not both contemporary and historical species lists were available for that compartment for each taxonomic group (VA = Vascular plants, NV = Non-vascular plants, OR = Orthoptera, AN = Ants, SY = Hoverflies and OD = Odonata). Habitat type (Heath_T = tertiary heath, Heath_D = dune heath, H_Shore = harbour shore), compartment size and British National Grid coordinates for compartment centroids also shown.

| **Compartment Name** | **c.n_VA** | **c.n_NV** | **c.n_OR** | **c.n_AN** | **c.n_SY** | **c.n_OD** | **Eco.habitat** | **cmpt.area_m^2** | **x_coord_BNG** | **y_coord_BNG** |
| --- | --- | --- | --- | --- | --- | --- | --- | --- | --- | --- |
| Brands heath | 1 | 1 | 1 | 1 | 1 | 1 | Heath_T | 72367.88 | 402378.5 | 84712.94 |
| Curlew cottages | 2 | 2 | NA | NA | NA | 2 | Heath_T | 899.5889 | 402577.2 | 85086.06 |
| Curlew heath | 3 | 3 | 3 | 3 | 3 | 3 | Heath_T | 74255.92 | 402704.5 | 85241.86 |
| Little sea enclosure | 4 | 4 | NA | NA | 4 | 4 | Heath_T | 10853.81 | 402690 | 84739.12 |
| Pipley heath | 5 | 5 | NA | NA | 5 | NA | Heath_T | 15024.35 | 402919.5 | 83773.62 |
| Plateau heath north | 6 | 6 | 6 | 6 | 6 | 6 | Heath_T | 97817.7 | 402884.5 | 85772.53 |
| Plateau heath south | 7 | 7 | 7 | 7 | 7 | 7 | Heath_T | 200764.4 | 402518.2 | 85397.56 |
| Spur heath | 8 | 8 | 8 | 8 | NA | 8 | Heath_T | 114452.2 | 402594.9 | 84582.95 |
| Western Arm Heath | 9 | 9 | 9 | 9 | 9 | 9 | Heath_T | 46662.21 | 402587.6 | 84965.55 |
| Wood Heath | 10 | 10 | NA | 10 | NA | NA | Heath_T | 6391.836 | 402853.3 | 84017.74 |
| East heath pines | 11 | NA | NA | NA | NA | 11 | Heath_D | 7744.334 | 403315.3 | 84887.3 |
| First ridge north | 12 | 12 | 12 | 12 | NA | 12 | Heath_D | 129864.2 | 403911.5 | 85617.07 |
| First ridge south | 13 | 13 | 13 | 13 | 13 | 13 | Heath_D | 172979.8 | 403478 | 84606.38 |
| Inner Ridge | 14 | 14 | NA | 14 | NA | 14 | Heath_D | 24413.98 | 403295.2 | 84417.08 |
| Pipley hollow | 15 | 15 | 15 | 15 | 15 | NA | Heath_D | 3204.267 | 403272.5 | 83961.11 |
| Second ridge N | 16 | 16 | 16 | 16 | 16 | 16 | Heath_D | 144635.4 | 403657.7 | 85675 |
| Second ridge S | 17 | 17 | 17 | 17 | NA | 17 | Heath_D | 29201.24 | 403411.7 | 85252.74 |
| Southern heath | 18 | 18 | 18 | 18 | 18 | 18 | Heath_D | 127788.7 | 403250.3 | 84240.32 |
| Third ridge central | 19 | 19 | 19 | 19 | NA | 19 | Heath_D | 67848.99 | 403334.6 | 85863.15 |
| Third ridge north | 20 | 20 | 20 | 20 | NA | 20 | Heath_D | 38697.82 | 403272.4 | 86066.33 |
| Third ridge south | 21 | NA | 21 | 21 | NA | 21 | Heath_D | 26285.42 | 403137.3 | 85339.74 |
| Third ridge west | 22 | 22 | 22 | 22 | NA | NA | Heath_D | 11127.94 | 403128.5 | 85555.2 |
| Third ridge west heath | 23 | 23 | NA | NA | NA | NA | Heath_D | 12711.96 | 403131.4 | 85727.11 |
| Aspen wood | 24 | 24 | NA | 24 | 24 | NA | Wood | 9446.228 | 403146.1 | 83713.37 |
| Northern enclosure | 25 | NA | NA | NA | NA | 25 | Wood | 24884.99 | 403561.7 | 86244.78 |
| Northern Enclosure Extension | 26 | 26 | NA | 26 | NA | 26 | Wood | 7066.84 | 403501 | 86283.17 |
| Pipley enclosure | 27 | 27 | 27 | 27 | 27 | 27 | Wood | 34921.55 | 403111.6 | 83743.02 |
| Pipley wood | 28 | 28 | 28 | 28 | NA | 28 | Wood | 30188.62 | 403194.8 | 83795.34 |
| Plateau enclosure | 29 | 29 | NA | 29 | 29 | 29 | Wood | 13318.97 | 403021.2 | 85607.95 |
| Plateau enclosure extension | 30 | 30 | 30 | 30 | NA | 30 | Wood | 7791.239 | 403001 | 85636.02 |
| Third ridge pines | 31 | 31 | NA | 31 | 31 | 31 | Wood | 7865.834 | 403044.4 | 85455.08 |
| Three acre wood | 32 | 32 | NA | 32 | NA | NA | Wood | 9910.083 | 402911.9 | 84003.27 |
| Twelve acre wood | 33 | 33 | NA | 33 | 33 | 33 | Wood | 50491.84 | 402914.2 | 84195.13 |
| Western Arm Pines | 34 | NA | NA | 34 | NA | 34 | Wood | 24283.05 | 402583 | 84743.82 |
| Knoll Dunes | 35 | NA | NA | NA | NA | NA | Dune | 4541.302 | 403391.7 | 83654.48 |
| Lone dune | 36 | NA | NA | 36 | NA | 36 | Dune | 5878.515 | 403243.5 | 84335.71 |
| Northern dunes | 37 | NA | 37 | NA | NA | 37 | Dune | 10973.43 | 403666.3 | 86262.08 |
| Shell bay dunes | 38 | 38 | 38 | 38 | 38 | 38 | Dune | 78455.08 | 403754.6 | 86154.26 |
| South haven flats | 39 | NA | NA | NA | 39 | NA | Dune | 6286.17 | 403451.5 | 86346.55 |
| Zero ridge south | 40 | NA | NA | NA | NA | NA | Dune | 104029.1 | 403608.7 | 84623.99 |
| Bramble bush bay | 41 | NA | 41 | 41 | NA | 41 | H_shore | 131970.6 | 403101.9 | 86109.75 |
| Brands creek | 42 | 42 | 42 | 42 | NA | 42 | H_shore | 26117.14 | 402276.8 | 84788.05 |
| Dyke bay | 43 | 43 | 43 | NA | NA | NA | H_shore | 76795.11 | 402703.1 | 85840.35 |
| Dyke bay saltmarsh | 44 | NA | NA | NA | NA | NA | H_shore | 6624.107 | 402531.3 | 85683.51 |
| Gravel Spit | 45 | NA | 45 | 45 | NA | NA | H_shore | 4574.459 | 402924.7 | 85982.3 |
| Plateau bay | 46 | NA | NA | NA | NA | NA | H_shore | 49603.62 | 402335.1 | 85642.19 |
| Redhorn bay ex ciffs | 47 | NA | 47 | 47 | NA | NA | H_shore | 65331.81 | 402255.8 | 85251.07 |
| Redhorn quay ex shore | 48 | 48 | NA | 48 | NA | NA | H_shore | 5514.588 | 402283.1 | 85493.83 |
| Sandy point | 49 | NA | NA | NA | 49 | NA | H_shore | 1170.44 | 402481 | 85708.04 |
| Central marsh north | 50 | 50 | 50 | 50 | 50 | 50 | Marsh | 131093.8 | 403488.1 | 85829.4 |
| Central marsh south | 51 | 51 | 51 | 51 | 51 | 51 | Marsh | 114065.2 | 403203.5 | 85228.98 |
| Central tongue | 52 | 52 | NA | 52 | NA | 52 | Marsh | 4295.939 | 403708.3 | 85316.46 |
| East marsh | 53 | 53 | 53 | 53 | 53 | 53 | Marsh | 64881.64 | 403228.7 | 84752.52 |
| Eastern lake marsh | 54 | 54 | 54 | 54 | 54 | 54 | Marsh | 89181.82 | 403394.1 | 85071.19 |
| Little sea swamp | 55 | NA | 55 | NA | NA | 55 | Marsh | 35265.17 | 402857 | 84740.26 |
| Lone dunes marsh | 56 | NA | NA | NA | NA | NA | Marsh | 1919.025 | 403203.6 | 84368.73 |
| New pool marsh | 57 | 57 | 57 | NA | 57 | 57 | Marsh | 49369.14 | 403076 | 85404.74 |
| Northern dunes marsh | 58 | NA | 58 | 58 | NA | NA | Marsh | 5918.71 | 403644.8 | 86198.01 |
| Northern tongues | 59 | NA | 59 | 59 | NA | NA | Marsh | 7408.769 | 403991.6 | 85868.52 |
| One Acre Pool Marsh | 60 | NA | 60 | 60 | 60 | 60 | Marsh | 22383.47 | 403204.4 | 85735.34 |
| Pipley swamp | 61 | 61 | 61 | 61 | 61 | 61 | Marsh | 57982.68 | 403011.1 | 83871.71 |
| Saltings strip | 62 | 62 | 62 | 62 | 62 | 62 | Marsh | 49044.66 | 403766.1 | 85581.13 |
| Spur bog | 63 | 63 | 63 | 63 | 63 | 63 | Marsh | 33773.25 | 402725.1 | 84294.74 |
| Western Arm Marsh | 64 | 64 | NA | NA | 64 | 64 | Marsh | 15544.56 | 402739.7 | 84998.22 |
| Wood Marsh | 65 | 65 | NA | NA | 65 | NA | Marsh | 3016.789 | 402843.8 | 83925.32 |
| Eastern lake | 66 | NA | NA | NA | NA | 66 | Aquatic | 25373.45 | 403469.7 | 85047.66 |
| Little sea central | 67 | NA | NA | NA | NA | 67 | Aquatic | 86303.72 | 402994.9 | 84876.4 |
| LS Northern one | 68 | NA | NA | NA | 68 | 68 | Aquatic | 6590.469 | 402984.4 | 85512.43 |
| LS Northern two | 69 | NA | NA | NA | 69 | 69 | Aquatic | 58613.8 | 402879.7 | 85235.29 |
| LS Southern one | 70 | NA | NA | NA | NA | 70 | Aquatic | 108148.2 | 402969 | 84469 |
| LS Southern two | 71 | NA | NA | 72 | NA | 71 | Aquatic | 41159.31 | 403082 | 84155.51 |
| One acre pool main pool | 72 | NA | NA | NA | NA | 72 | Aquatic | 4465.494 | 403237.4 | 85831.78 |
| Pipley pools | 73 | NA | 73 | 73 | 73 | 73 | Aquatic | 9825.182 | 403250 | 83748.87 |
| Western Arm | 74 | NA | NA | NA | 74 | 74 | Aquatic | 29835.04 | 402748.4 | 84844.74 |

## S2 | The Models

${SGL}_{i}$ ~Bernoulli ${(p}_{i})$

Logit ${(p}_{i})$ = $A_{i}^{VA}$ + $B_{i}^{NV}$ $B_{i}^{OR}$+ $B_{i}^{AN}$ + $B_{i}^{SY}$ + $B_{i}^{OD}$ +${Sp}_{species i}$

$A_{i}^{VA}$ = α + $\alpha_{cmpt i}$ $\sigma_{\alpha cmpt}$

$B_{i}^{NV}$ = $\beta^{NV}$ + $\beta_{cmpt i}^{NV}$ $\sigma_{\beta cmpt}^{NV}$

$B_{i}^{OR}$ = $\beta^{OR}$ + $\beta_{cmpt i}^{OR}$ $\sigma_{\beta cmpt}^{OR}$

$B_{i}^{AN}$ = $\beta^{AN}$ + $\beta_{cmpt i}^{AN}$ $\sigma_{\beta cmpt}^{AN}$

$B_{i}^{SY}$ = $\beta^{SY}$ + $\beta_{cmpt i}^{SY}$ $\sigma_{\beta cmpt}^{SY}$

$B_{i}^{OD}$ = $\beta^{OD}$ + $\beta_{cmpt i}^{OD}$ $\sigma_{\beta cmpt}^{OD}$

$\left( \begin{aligned} \alpha_{cmpt} \\ \beta_{cmpt}^{NV} \\ \beta_{cmpt}^{OR} \\ \beta_{cmpt}^{AN} \\ \beta_{cmpt}^{SY} \\ \beta_{cmpt}^{OD} \end{aligned} \right)$ ~ MVNormal ${(0,S}_{cmpt})$

${Sp}_{species i}$ ~ Normal $(0, \sigma_{species})$

(α, $\beta^{NV}$, $\beta^{OR}$, $\beta^{AN}$, $\beta^{SY}$, $\beta^{OD}$) ~ Normal (0, 5)

($\sigma_{\alpha cmpt}$, $\sigma_{\beta cmpt}^{NV}$, $\sigma_{\beta cmpt}^{OR}$, $\sigma_{\beta cmpt}^{AN}$, $\sigma_{\beta cmpt}^{SY}$, $\sigma_{\beta cmpt}^{OD}$) ~ HalfCauchy(0, 2.5)

$\sigma_{species}$ ~ HalfCauchy(0, 2.5)

Above is an algebraic representation of model M1. The model is parameterised with an intercept $A_{i}^{VA}$ which estimates the proportion of vascular plant species gains vs losses. $A_{i}^{VA}$ varies by sampling compartment and is broken down into a mean (α) and compartment offset in the sub-model ($A_{i}^{VA}$ = α + $\alpha_{cmpt i}$ $\sigma_{\alpha cmpt}$), where $\sigma_{\alpha cmpt}$ provides an estimate of the variance among compartments. There are five sets of similarly varying slope parameters ($B_{i}^{NV},$ $B_{i}^{OR},$ $B_{i}^{AN}$,$B_{i}^{SY}$ and $B_{i}^{OD}$), each of which estimates the difference in the proportion of species gains vs losses by compartment between the vascular plants and one of the remaining five focal taxa (non-vascular plants, Orthoptera, ants, hoverflies and Odonata). To construct intercept for any of these five taxa, we simply add the vascular plant intercept ($A_{i}^{VA}$ ) and the relevant slope value for the taxa of interest (eg. $A_{i}^{VA}$ + $B_{i}^{SY}$ to construct intercepts for the hoverflies). To get a measure of pairwise congruencies in local changes (gains vs losses) between taxa across the peninsula, we construct posterior intercepts for each taxon and compute Pearson’s correlations between them for each pair across the entire posteriors.

We now have pairwise distributions of correlations between taxa. However, we don’t know what’s causing these correlations; is it shared responses to environmental change, or possibly biotic interactions between taxa? We try to shed some light on the answer to this question by adjusting for the effects of local changes in the environment on proportions of species gains vs losses and recomputing the correlations.

We present model M2 with environmental predictors in the R code used to fit the model, rather than expressing it algebraically:

M2 <- map2stan(

alist(

# ikelihood

LG ~ dbinom(1,p),

# linear models

logit(p) <- (A + B_NV*NV + B_OR*OR + B_AN*AN + B_SY*SY + B_OD*OD) + Sp_offset[Species],

A <- a + a_Cmpt[Cmpt] + a_Hab[Hab] + ba_F[Hab]*EIV_F + ba_L[Hab]*EIV_L + ba_R[Hab]*EIV_R + ba_S[Hab]*EIV_S,

B_NV <- bnv + bnv_Cmpt[Cmpt] + bnv_Hab[Hab] + bnv_F[Hab]*EIV_F + bnv_L[Hab]*EIV_L + bnv_R[Hab]*EIV_R + bnv_S[Hab]*EIV_S,

B_OR <- bor + bor_Cmpt[Cmpt] + bor_Hab[Hab] + bor_F[Hab]*EIV_F + bor_R[Hab]*EIV_R,

B_AN <- ban + ban_Cmpt[Cmpt] + ban_Hab[Hab] + ban_F[Hab]*EIV_F + ban_L[Hab]*EIV_L + ban_S[Hab]*EIV_S,

B_SY <- bsy + bsy_Cmpt[Cmpt] + bsy_Hab[Hab] + bsy_F[Hab]*EIV_F + bsy_L[Hab]*EIV_L,

B_OD <- bod + bod_Cmpt[Cmpt] + bod_Hab[Hab] + bod_F[Hab]*EIV_F + bod_L[Hab]*EIV_L + bod_R[Hab]*EIV_R + bod_S[Hab]*EIV_S,

# adaptive priors

c(a_Cmpt,bnv_Cmpt,bor_Cmpt,ban_Cmpt,bsy_Cmpt,bod_Cmpt)[Cmpt] ~ dmvnormNC(sigma_Cmpt,Rho_Cmpt),

c(a_Hab,bnv_Hab,bor_Hab,ban_Hab,bsy_Hab,bod_Hab,

ba_F, bnv_F, bor_F, ban_F, bsy_F, bod_F,

ba_L, bnv_L, ban_L, bsy_L, bod_L,

ba_R, bnv_R, bor_R, bod_R,

ba_S, bnv_S, ban_S, bod_S)[Hab] ~ dmvnormNC(sigma_Hab,Rho_Hab),

Sp_offset[Species] ~ dnorm(0,sigma_Sp),

# fixed priors

c(a,bnv,bor,ban,bsy,bod) ~ dnorm(0,5),

sigma_Cmpt ~ dcauchy(0,2.5),

sigma_Hab ~ dcauchy(0,2.5),

sigma_Sp ~ dcauchy(0,2.5),

Rho_Cmpt ~ dlkjcorr(1),

Rho_Hab ~ dlkjcorr(2)

) , data=df , iter=4000, warmup=1000, chains=4, cores=2)

Now the sub-model for the vascular plant intercept is further broken down to include a variable offset for the habitat type a compartment belongs to, and varying slopes for mean changes per compartment in Ellenberg F, L, R, and S values (specifying wetness, light availability, pH, and salinity respectively). To get an estimate of the proportional species gains vs losses after controlling for these factors, we just add the mean and compartment offset (α + $\alpha_{cmpt i}$). We similarly account for any systematic differences between the other taxa and the vascular plant attributable to these factors by adding them as predictors in the sub-models for the slope parameters. We then recompute the posterior correlations having controlled for responses to these environmental changes (eg. correlation between vascular plants and hoverflies is the correlation between (α + $\alpha_{cmpt i}$) and (α + $\alpha_{cmpt i}$+ $\beta^{SY}$ + $\beta_{cmpt i}^{SY}$)).

Figure S2 Parameter estimates from sub-model predictors of model M2

## S3 | Species Accumulation Curves (2013-2015)

Species accumulation curves (SACs) were fitted for the 2010s sampling period across sampling instances during which the presence of at least one species for a given taxonomic group was recorded. SCAs were compile using the “specaccum” function from the R package “vegan” (Oksanen et al. 2013). It should be noted that these curves are likely to be quite conservative. There may have been many more visits to particular sampling compartments during which no species that were newly recorded in that particular compartment were found. Recording of this type of visit was not consistent across all taxa and recorders; often repeat records of species for a given compartment were not recorded, and thus if no new species was found the visit was effectively unrecorded. As such, the number of actual visits will often have been larger in reality than displayed below on habitat-specific SACs, and to a much lesser extent for whole peninsula SACs, for taxonomic groups. This bias may be pronounced for ‘species poor’ habitats. SACs are displayed below in Figures S3-S10.


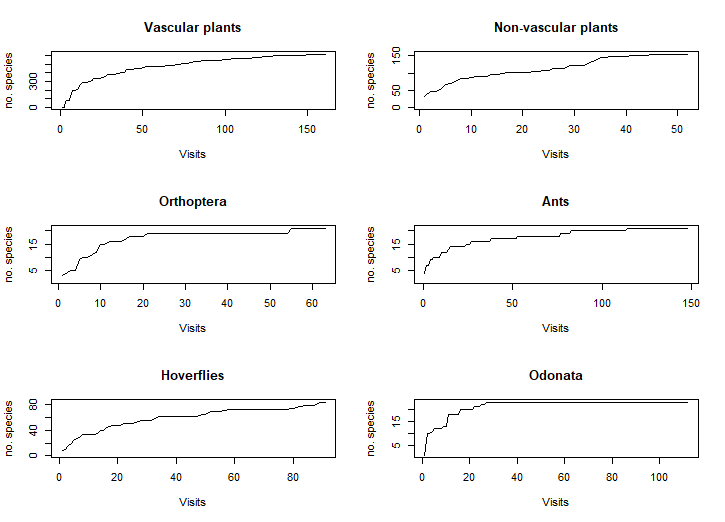


Figure S3 Whole Peninsula level Species Accumulation Curves 2013-15


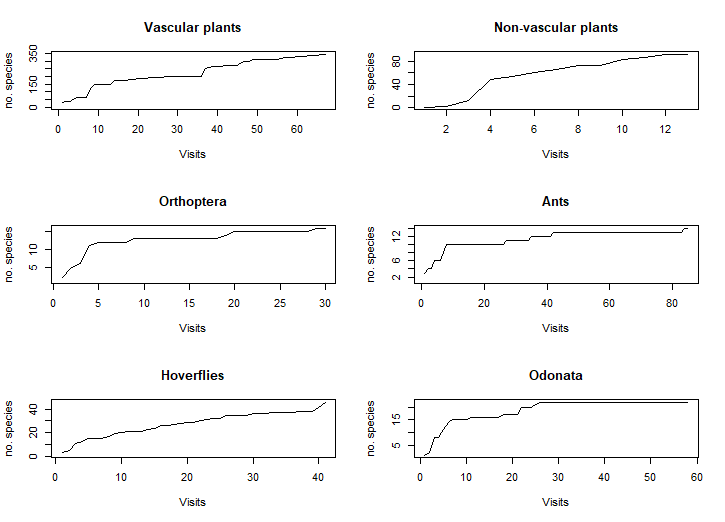


Figure S4 Heath level Species Accumulation Curves 2013-15


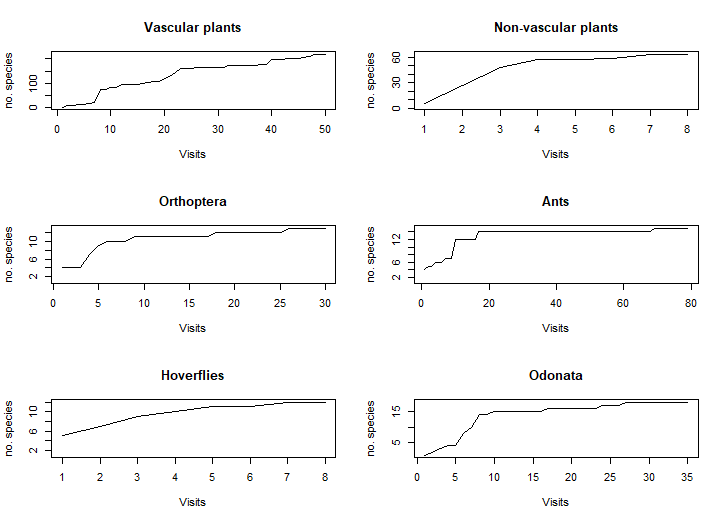


Figure S5 Dune Heath level Species Accumulation Curves 2013-15


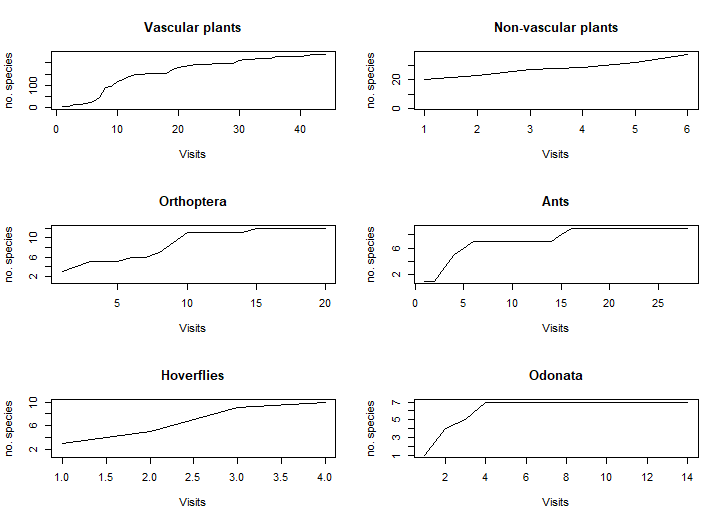


Figure S6 Dune level Species Accumulation Curves 2013-15


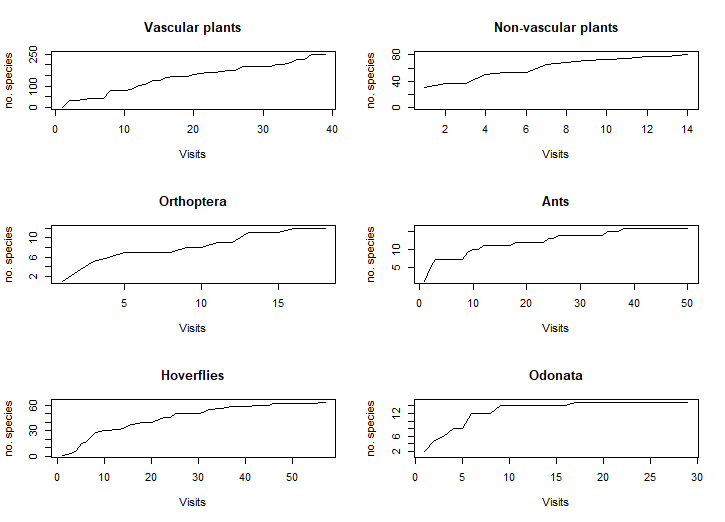


Figure S7 Woods level Species Accumulation Curves 2013-15


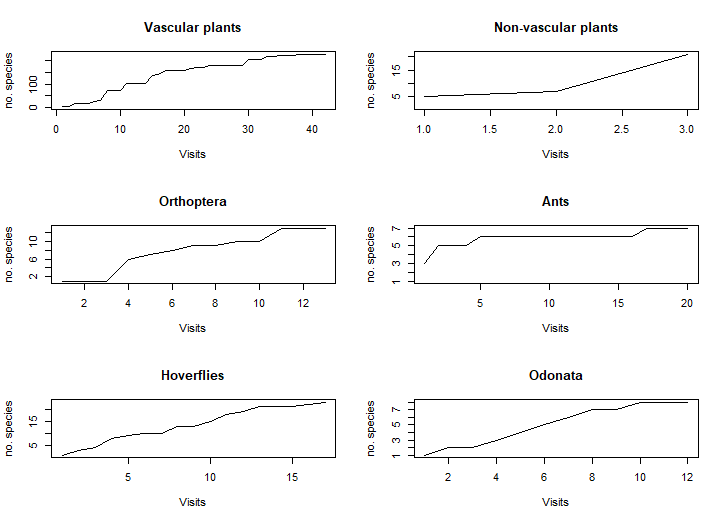


Figure S8 Harbour Shore level Species Accumulation Curves 2013-15


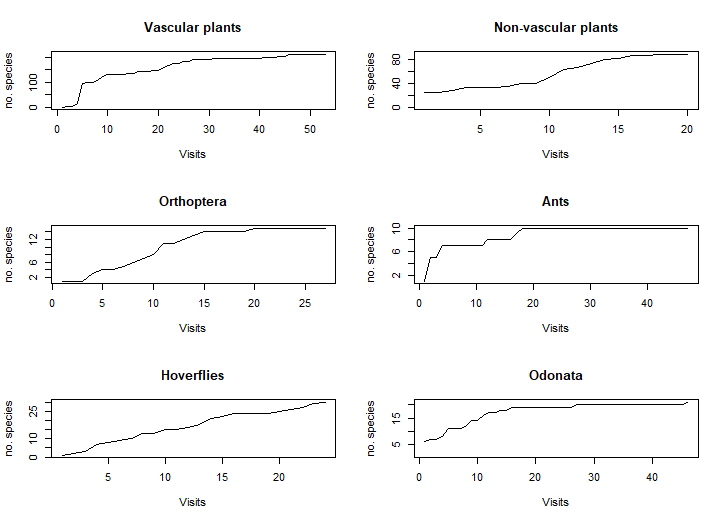


Figure S9 Marsh level Species Accumulation Curves 2013-15


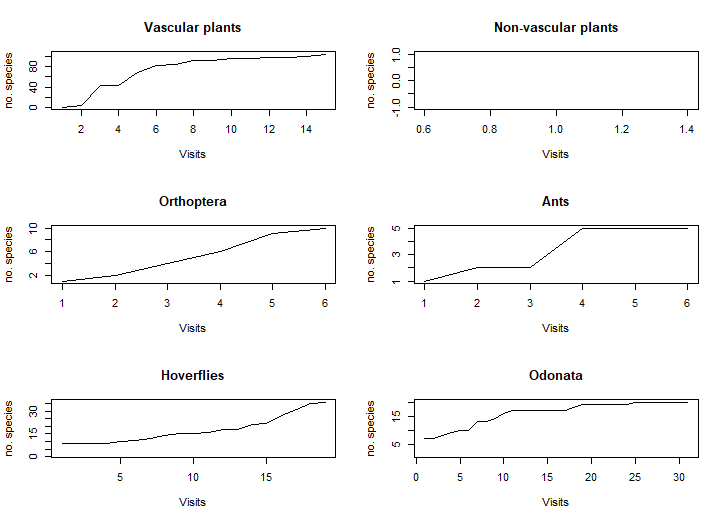


Figure S10 Aquatic level Species Accumulation Curves 2013-15

## S4 | Raup-Crick Null Models

Within taxonomic groups, the sampling compartments of Studland vary considerably in terms of alpha diversity (species richness), which is likely at least partially due to differing sizes of sampling compartments. Differential compartment sizes could conceivably affect estimates of community composition, and as a result, estimates of compositional differences across time-periods, for a few different reasons. For example, compartment size could simultaneously affect compartment-specific species richness and sampling effort, as well as stochastic processes (i.e., drift) which determine the likelihood of species losses and/or gains. As such, there is a potential danger that observed differences in community composition within sampling compartments (across time-periods) could emerge due to chance or to confounding effects of compartment size. To mitigate for this possibility, we used a “Raup Crick” based Null model approach to assess whether observed differences in community composition across time-periods (i.e., temporal beta diversity) are likely to have occurred by chance (Chase et al. 2011).

The Raup-Crick (RC) metric ranges from -1 to 1, indicating whether local communities are more dissimilar (approaching 1), as dissimilar (approaching 0), or less dissimilar (approaching -1), than would be expected by random chance (Chase et al. 2011), while accounting for potential biases introduced due to levels of alpha and gamma diversity. We computed RC differences between all pairs of sampling compartments across both sampling periods for each taxonomic group, with gamma diversity (conservatively) compiled from all species recorded on the peninsula across both time-periods. We were particularly interested in the specific paired differences in community composition estimated within compartments across sampling periods, and possible effects of compartment size on these differences.

We found no evidence of compartment size on RC differences between time-periods (Figure S11). For four taxonomic groups (vascular and non-vascular plants, Orthoptera and Odonata), community composition within compartments across time-periods, as estimated by RC differences, skewed towards being more similar than would be expected by chance and more similar than comparisons across sampling compartments (Figure S12). This suggests that between time-period comparisons across datasets were indeed meaningful, as this signal would be expected if some ‘core’ of local species often remained in a given area between sampling periods. Of the two other taxa, for the ants, within compartments (across time-period) RC estimates were approximately equally likely to be more or less similar to what would be expected by chance than were estimates across compartments, and the hoverflies skewed towards being somewhat more different than one would expect by chance (Figure S12). Possible reasons for these discrepancies are discussed in the main text.


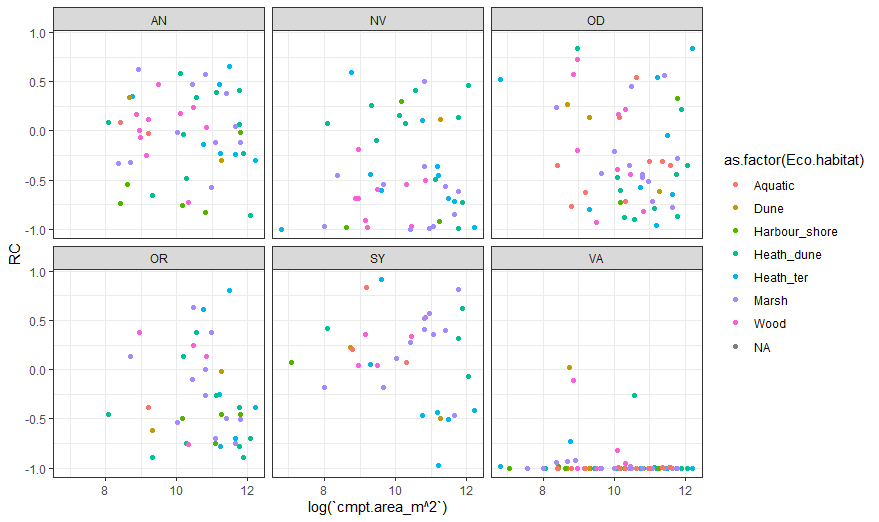


Figure S11 Log area of sampling compartments plotted against Raup-Crick dissimilarity estimates for within sampling compartment comparisons between time-periods show no effect of the size of sampling compartments on whether between time-period dissimilarities were greater or less than expected relative to the Null expectation.


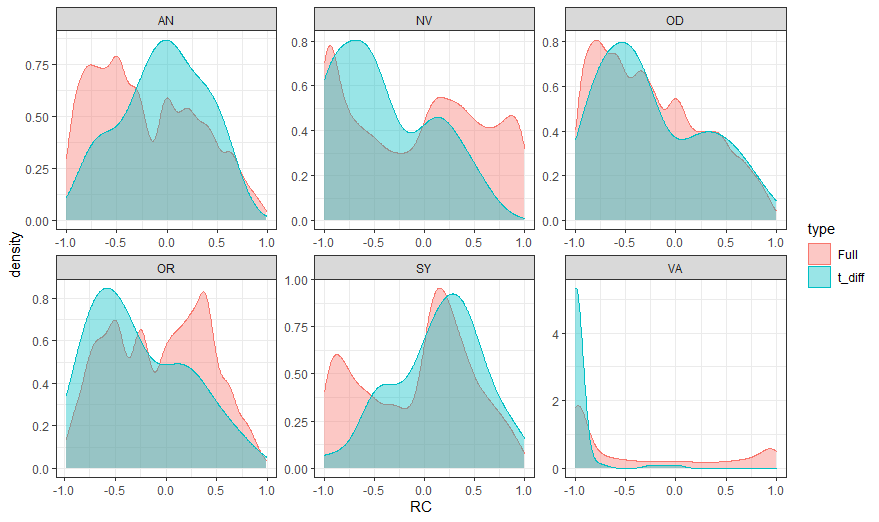


Figure S12 Density plots of Raup-Crick (RC) dissimilarity metrics for i) all sampling compartments for both time-periods combined (salmon colour), and ii) just between time-period comparisons within sampling compartments (turquoise). RC value of -1 for the dissimilarity between a pair of assemblages means that the assemblages are much more similar than would be expected by random chance, given the species pool and alpha diversity of assemblages. RC value of 1 means that the assemblages are much more different than would be expected by random chance, given the species pool and alpha diversity of assemblages. RC value of 0 means assemblages are as different as would be expected by random chance, given the species pool and alpha diversity of assemblages (Chase et al 2011).

## 5 | Spatial Autocorrelation

We tested for spatial autocorrelation in “sampling compartment” level proportional species gain vs losses for each taxonomic group, for model estimates of the same proportions, and for compartment-level model residuals from hierarchical logistic regression models. We computed autocorrelation in R by computing Moran’s I values on an adjacency matrix of sampling compartments following (Brunsdon and Comber 2018). The adjacency matrix is a matrix indicating whether each sampling compartment pair share a boundary.

Min and max possible values of Moran’s I, given W-matrix for compartments for each taxa are:

VA: [1] -1.082229 1.230397; NV:[1] -1.036326 1.104035; OR: [1] -1.031874 1.113671; AN: [1] -1.223380 1.278873; SY: [1] -1.154701 1.154701; OD: [1] -1.039250 1.133289

Table S2 Table of Moran’s I with associated p-values for raw mean species gains vs losses at sampling compartment level for vascular plants (VA), non-vascular plants (NV), Orthoptera (OR), ants (AN), hoverflies (SY) and Odonata (OD).

|  | VA | NV | OR | AN | SY | OD |
| --- | --- | --- | --- | --- | --- | --- |
| *Moran’s I:* | 0.39 | -0.156 | 0.123 | 0.149 | 0.23 | -0.105 |
| *p-value:* | < 0.005 | 0.85 | 0.15 | 0.09 | 0.06 | 0.79 |

Table S3 Table of Moran’s I with associated p-values for model estimates of proportional species gains vs losses at sampling compartment level for vascular plants (VA), non-vascular plants (NV), Orthoptera (OR), ants (AN), hoverflies (SY) and Odonata (OD). Models are Hierarchical Logistic Regression models M1 and M2 outlined in Section 1 above.

| Model |  | VA | NV | OR | AN | SY | OD |
| --- | --- | --- | --- | --- | --- | --- | --- |
| **M1** | *Moran’s I:* | 0.4 | -0.145 | 0.234 | 0.176 | 0.39 | -0.061 |
|  | *p-value:* | < 0.005 | 0.85 | 0.04 | 0.06 | 0.005 | 0.65 |
| **M2** | *Moran’s I:* | 0.39 | -0.15 | 0.242 | 0.212 | 0.412 | -0.088 |
|  | *p-value:* | < 0.005 | 0.84 | 0.03 | 0.03 | 0.004 | 0.74 |

Table S4 Table of Moran’s I with associated p-values for binned model residuals for proportional species gains vs losses at sampling compartment level for vascular plants (VA), non-vascular plants (NV), Orthoptera (OR), ants (AN), hoverflies (SY) and Odonata (OD). Models are Hierarchical Logistic Regression models M1 and M2 outlined in Section 1 above.

| Model |  | VA | NV | OR | AN | SY | OD |
| --- | --- | --- | --- | --- | --- | --- | --- |
| **M1** | *Moran’s I:* | 0.08 | -0.088 | -0.078 | 0.169 | -0.223 | -0.105 |
|  | *p-value:* | 0.13 | 0.71 | 0.64 | 0.06 | 0.88 | 0.79 |
| **M2** | *Moran’s I:* | 0.187 | -0.077 | -0.074 | 0.179 | -0.511 | -0.06 |
|  | *p-value:* | 0.009 | 0.67 | 0.63 | 0.05 | 0.99 | 0.65 |

Vascular plants, ants and hoverflies showed distinct patterns of autocorrelation in both compartment-level mean observations of proportional species gain vs losses, and model estimates of gains vs losses. Spatial autocorrelation for raw estimates of gains vs losses was only statistically significant for vascular plants (Table S2), but modelled estimates of the same were also statistically significant for Orthoptera and hoverflies for estimates from model M1, and for Orthoptera, ants and hoverflies for estimates from model M2 (Table S3). However, statistically significant spatial autocorrelation was only apparent in compartment-level binned model residuals for vascular plants under the model including habitat and EIV predicters (though the p-value for ants was also borderline at 0.05; Table S4). No other model residuals displayed evidence of spatial autocorrelation. Despite evidence of autocorrelation in the raw data, the lack of spatially autocorrelated residuals in hierarchical logistic regression models alleviates any potential concern of biased model estimates that could affect our finding of cross-taxon congruence in local species richness change (Kühn and Dormann 2012).

## S6 | Posterior predictive checks

Figure S13 PPcheck for model m0 without the species offset.

Figure S14 PPcheck for model M1.

Adding the species offset does introduce uncertainty into the compartment level estimates as we would expect – particularly for Orthoptera and ant groups. However, they are still mostly congruent with the raw proportions.

Figure S15 PPcheck for model M2 with environmental predictors.

Again, adding the sub-model predictors causes a little more uncertainty in the compartment level estimates, but they are still mostly in agreement with the raw proportions.

## 7 | References

Brunsdon, C. and Comber, L., 2018. *An introduction to r for spatial analysis and mapping*. 2nd edition. Thousand Oaks, CA: SAGE Publications.

Chase, J. M., Kraft, N. J. B., Smith, K. G., Vellend, M. and Inouye, B. D., 2011. Using null models to disentangle variation in community dissimilarity from variation in α-diversity. *Ecosphere*, 2 (2), art24.

Kühn, I. and Dormann, C. F., 2012. Less than eight (and a half) misconceptions of spatial analysis: Correspondence. *Journal of Biogeography*, 39 (5), 995–998.

Legendre, P. and Legendre, L. F. J., 2012. *Numerical Ecology*. Elsevier.

Oksanen, J., Blanchet, F. G., Kindt, R., Legendre, P., Minchin, P. R., O’Hara, R. B., Simpson, G. L., Solymos, P., Henry, Stevens, H. and Wagner, H., 2013. vegan: Community Ecology Package. R package version 2.0-10. http://CRAN.R-project.org/package=vegan.
